# A role for human brain pericytes in neuroinflammation

**DOI:** 10.1186/1742-2094-11-104

**Published:** 2014-06-11

**Authors:** Deidre Jansson, Justin Rustenhoven, Sheryl Feng, Daniel Hurley, Robyn L Oldfield, Peter S Bergin, Edward W Mee, Richard LM Faull, Mike Dragunow

**Affiliations:** 1 Department of Pharmacology and Clinical Pharmacology, The University of Auckland, 85 Park Road, Auckland 1023, New Zealand; 2 Gravida National Centre for Growth and Development, The University of Auckland, Bldg 505, 85 Park Road, Auckland 1023, New Zealand; 3 Department of Anatomy with Radiology, The University of Auckland, Bldg 505, 85 Park Road, Auckland 1023, New Zealand; 4 Centre for Brain Research, The University of Auckland, Bldg 503, 85 Park Road, Auckland 1023, New Zealand; 5 Department of Molecular Medicine and Pathology, The University of Auckland, Bldg 504, 85 Park Road, Auckland 1023, New Zealand; 6 LabPLUS, Auckland City Hospital, Bldg 31, Gate 4 Grafton Road, Auckland 1148, New Zealand; 7 Auckland City Hospital, 2 Park Rd, Auckland 1010, New Zealand

**Keywords:** Microglia, Astrocytes, Inflammation, Blood–brain barrier, Chemokines

## Abstract

**Background:**

Brain inflammation plays a key role in neurological disease. Although much research has been conducted investigating inflammatory events in animal models, potential differences in human brain versus rodent models makes it imperative that we also study these phenomena in human cells and tissue.

**Methods:**

Primary human brain cell cultures were generated from biopsy tissue of patients undergoing surgery for drug-resistant epilepsy. Cells were treated with pro-inflammatory compounds IFNγ, TNFα, IL-1β, and LPS, and chemokines IP-10 and MCP-1 were measured by immunocytochemistry, western blot, and qRT-PCR. Microarray analysis was also performed on late passage cultures treated with vehicle or IFNγ and IL-1β.

**Results:**

Early passage human brain cell cultures were a mixture of microglia, astrocytes, fibroblasts and pericytes. Later passage cultures contained proliferating fibroblasts and pericytes only. Under basal culture conditions all cell types showed cytoplasmic NFκB indicating that they were in a non-activated state. Expression of IP-10 and MCP-1 were significantly increased in response to pro-inflammatory stimuli. The two chemokines were expressed in mixed cultures as well as cultures of fibroblasts and pericytes only. The expression of IP-10 and MCP-1 were regulated at the mRNA and protein level, and both were secreted into cell culture media. NFκB nuclear translocation was also detected in response to pro-inflammatory cues (except IFNγ) in all cell types. Microarray analysis of brain pericytes also revealed widespread changes in gene expression in response to the combination of IFNγ and IL-1β treatment including interleukins, chemokines, cellular adhesion molecules and much more.

**Conclusions:**

Adult human brain cells are sensitive to cytokine challenge. As expected ‘classical’ brain immune cells, such as microglia and astrocytes, responded to cytokine challenge but of even more interest, brain pericytes also responded to such challenge with a rich repertoire of gene expression. Immune activation of brain pericytes may play an important role in communicating inflammatory signals to and within the brain interior and may also be involved in blood brain barrier (BBB) disruption . Targeting brain pericytes, as well as microglia and astrocytes, may provide novel opportunities for reducing brain inflammation and maintaining BBB function and brain homeostasis in human brain disease.

## Introduction

Brain inflammation occurs in a number of neurological (for example, epilepsy, Alzheimer’s disease, Parkinson’s disease, motor neuron disease) and psychiatric (schizophrenia, depression) diseases and is generally thought to worsen disease symptoms and progression [1]. Although many different brain cells are likely to be involved in brain inflammation, most attention has focused on the role of non-nerve brain cells, especially microglia and astrocytes [2-4]. More recently, there has been an appreciation that perivascular cells may also be involved in brain inflammation. In particular, brain pericytes which surround endothelial cells and are critical in the development and maintenance of the blood brain barrier (BBB) may be targets as well as effectors of brain inflammatory processes [5,6]. Pericytes interact dynamically with astrocytes and microglia and can also receive signals from the periphery resulting in central nervous system (CNS) inflammatory molecule production [7,8].

CNS pericytes are also in a pivotal position to mediate interactions between systemic and central brain inflammation [9] and have been shown to play a role in recruitment of peripheral immune cells to the brain [10-12]. The further increase in brain infiltration of systemic immune molecules and cells as well as other blood components may directly cause neuronal damage and/or promote microglial inflammation [13].

For the above reasons, targeting systemic and central brain inflammatory pathways for treating brain disorders is a high priority for neuropharmacological drug development. Most of this work has been undertaken using animal models and given potential species differences more work needs to be undertaken to identify pathways specifically involved in human brain inflammation [14,15]. In this regard, mixed *in vitro* cultures from adult human brain tissue have been characterized by several groups [16-18]. However, until recently, the exact composition of these cultures has been unclear. Previous work in our group has revealed that a mixed population of astrocytes, microglia and fibroblast-like cells can be generated from adult human brain cultures [17,19]. More recently, we and others have identified a proportion of the fibroblast-like cells as human brain pericytes [5,19,20]. Many previous *in vitro* models of the BBB involve the use of animal or transformed human pericytes and brain microvascular endothelial cells [21]. We sought to investigate the effects of inflammatory molecules on primary adult human brain-derived cells (pericytes, astrocytes and microglia) that are in key locations for communicating inflammatory signals to and within the brain.

## Methods

### Reagents

DMEM/F12, fetal bovine serum (FBS) and PenStrep glutamine (PSG) were obtained from Gibco/Life Technologies (Carlsbad, CA, USA). Other reagents included human recombinant IFNγ (R&D Systems, Minneapolis, MN, USA), IL-1β, TNFα (PeproTech, Rocky Hill, NJ, USA), Triton™ X-100, Tween® 20, chloroform, lipopolysaccharide (LPS) and Hoechst 33528, and ExtrAvidin® -peroxidase (Sigma, St. Louise, MO, USA), TRIzol® (Ambion/Life Technologies, Carlsbad, CA, USA) and enhanced chemiluminescence (ECL) detection reagents (Amersham/GE Healthcare, Buckinghamshire, England).

### Biopsy brain tissue

Human middle temporal gyrus was obtained, with informed consent, from surgeries of patients with drug-resistant temporal lobe epilepsy, and with the approval of the Northern Regional Ethics Committee (New Zealand).

### Isolation of mixed glial cultures from human brain tissue

Brain tissue from the middle temporal gyrus (MTG), obtained following epilepsy surgery, was processed for the isolation and culture of microglia, astrocytes and brain pericytes, as previously described [19]. Cells were incubated at 37°C with 5% CO_2_ until seeding for experiments, approximately four to five days. Once mixed cultures reached the desired confluency, flasks were trypsinized with 0.25% trypsin-ethylenediaminetetraacetic acid (EDTA) and scraped to obtain microglia and astrocytes. Cells were seeded into 96-well plates at 5,000 cells/well in complete media (DMEM/F12 with 10% FBS and 1% PSG (penicillin 100 U/ml, streptomycin 100 μg/ml, L-glutamine 0.29 mg/ml)) and were used for experiments one to three days later. Experiments performed on mixed glial cultures were at passage 2 for all experiments in this study.

For further culture of primary cells, cells from passage 5 up to passage 9 were microglia- and astrocyte-free cultures and are referred to as pericytes throughout the text [17]. These cells were kept in complete media and were used directly or frozen in 5% dimethyl sulfoxide (DMSO), 95% FBS for later culture. Although many rodent studies derive pericytes from isolated blood vessels, consistent with our work, human brain studies have generally derived pericytes from dissociated grey matter [5,20].

Leptomeningeal explant cultures were obtained by removing meninges from MTG brain tissue and rinsing with complete media. Meninges were cut into small pieces, 2 to 3 mm^2^, and put into six-well plates containing 850 μl media/well, four to five pieces/well, and incubated at 37°C, 5% CO_2_ in complete media. Tissue was cultured with such low volumes of media so that it would remain stationary in the well. Media was changed after 48 to 72 hours in culture and every three to four days afterwards. Depending on cases, normally around one to two weeks in culture, cells began to migrate out from explants. After two to three weeks in culture, explants were moved to a new six-well plate to continue culture. Cells were generated from the explants again. This procedure was continued and explants were moved once underlying cells became confluent.

### Cell treatments

Cells were treated for the indicated times with IFNγ, IL-1β and LPS, each at 10 ng/ml, TNFα at 50 ng/ml, unless otherwise stated, or vehicle (0.1% BSA in phosphate buffered saline (PBS)).

### Immunocytochemistry

At endpoint, cells were fixed using 4% paraformaldehyde solution. After washing in PBS with 0.2% Triton X-100™ (PBS-T), plates were incubated with primary antibodies overnight at 4°C (all antibodies were diluted in goat immunobuffer (1% goat serum, 0.2% Triton X-100™, and 0.04% thiomersal in PBS)). Dilutions of antibodies are listed in Additional file 1: Table S1. Plates were washed again in PBS-T and incubated with secondary antibodies two to three hours at room temperature, then rinsed. For fluorescent staining, nuclei were detected using Hoechst stains. Otherwise, plates were incubated with ExtrAvidin-peroxidase diluted 1/500 in goat immunobuffer for one hour at room temperature then detected using 3,3’-diaminbenzidine. Discovery-1 Automated Fluorescence Microscope (Molecular Devices, Sunnyvale, CA, USA) or ImageXpress® Micro XLS (Version 5.3.0.1, Molecular Devices) housed at the Biomedical Imaging Research Unit, University of Auckland was used for image acquisition from micro-well plates [22]. Quantitative analysis of positively stained cells and total cells was performed using Metamorph® software (Version 6.2.6, Molecular Devices) [23] using the Cell Scoring module for interferon inducible protein 10 (IP-10) and monocyte chemotactic protein-1 (MCP-1) expression or MetaXpress® software (Version 5.3.0.1, Molecular Devices) for Multiwavelength Cell Scoring of co-labelling. Each condition was done in triplicate and four images/well were analyzed, with approximately 250 to 400 cells/field counted.

### Western blot

At endpoint, cells were rinsed with PBS and scraped into Eppendorf tubes. Cells were centrifuged and the pellet was resuspended in lysis buffer (25 mM Tris–HCl pH 7.5, 150 mM NaCl, 50 mM NaF, 0.5 mM EDTA pH 8, 0.5% Triton-X 100™, 5 mM β-glycerophosphate, with fresh 1 mM dithiothreitol (DTT), 1 mM phenylmethanesulfonyl fluoride (PMSF), 1 mM Na_3_VO_4_). A total of 10 to 20 μg protein diluted in Laemmli buffer (125 mM Tris–HCl, pH 6.8, 5% glycerol, 4% sodium dodecyl sulfate (SDS), 0.2% bromophenol blue) was separated on 4% to 12% pre-cast gels (Life Technologies) and separated by SDS-polyacrylamide gel electrophoresis (SDS-PAGE). Media samples for Western blot were collected and centrifuged to remove debris. Supernatants were transferred to new tubes and diluted in Laemmli buffer and separated on SDS-PAGE as above.

Fluorescent western blots were carried out as previously described [24]. Briefly, proteins were transferred to polyvinylidene difluoride (PVDF) membranes from Millipore (Billerica, MA, USA)(IPFL00010 Immobilon-FL 0.45 mm) for optimal fluorescence signal and blocked in Odyssey Blocking Buffer (Li-COR 927–40000) diluted 1:1 in Tris buffered saline with 0.1% Tween®-20 (TBS-T), for one hour at room temperature. Membranes were incubated with primary antibodies [see Additional file 1: Table S1] diluted in Odyssey Blocking Buffer and TBS-T (1:1) overnight at 4°C. Membranes were incubated with secondary antibodies from Li-COR diluted in Odyssey and TBS-T (1:1) with 0.1% Tween®-20 and 0.02% SDS for two hours at room temperature. Images were captured using the Li-COR imaging system. Conversely, for chemiluminescent western blots the signal was detected using ECL detection reagents (Amersham) and visualized using the Bio-Rad ChemiDoc™ imaging system.

### Quantitative RT-PCR

Quantitative reverse transcriptase polymerase chain reaction (qRT-PCR) was performed as described previously [19]. At endpoint, cells were rinsed 2× with PBS then TRIzol® (1 ml/well) was added and lysates were transferred to screw cap tubes. Chloroform (200 μl) was added and tubes were shaken to mix. Samples were centrifuged, the aqueous layer was transferred to a new tube and an equal volume of 70% ethanol (EtOH) was added. RNA was then extracted using RNeasy kits (Qiagen Inc. Venlo, Limburg, Nethlands). cDNA was made from 3μg DNase-1 (Promega, Madison, WI, USA) treated RNA using the Superscript III first strand synthesis kit (Invitrogen, Calrsbad, CA, USA). qRT-PCR was performed using Platinum SYBR Green qPCR SuperMix-UDG with Rox kit (Invitrogen). Standard curves were performed for all primers used; sequences and efficiencies are included in Additional file 2: Table S2. The level of gene expression was normalized to GAPDH at time zero or untreated conditions using the ΔC_t_ method [25].

### Microarray experiment

Five cases from epilepsy tissue were chosen for this experiment, all male, with an average age of 45 +/−7 years, with varying degrees of mesial temporal sclerosis. All cultures were frozen down in 5% DMSO in FBS at passage 3 or 4 after initial isolation. Cells were thawed from liquid nitrogen quickly at 37°C. Cells were transferred to 15 ml conicals with fresh pre-warmed complete DMEM/F12 and centrifuged to pellet cells. Cells were resuspended in 15 ml complete DMEM/F12 and transferred to T75 tissue culture flasks. Cells were incubated for three days to enable 80% to 90% confluency to be reached. Cells were trypsinized as indicated for human brain cell tissue culture and seeded (passage 5) into six-well plates at 1.37 × 10^5^ cells/well in complete DMEM/F12 and incubated at 37°C, 5% CO_2_ for two days. Cells were treated with vehicle (0.1% BSA in PBS) or IFNγ and IL-1β at a final concentration of 10 ng/ml for 24 hours, three wells of a six-well plate per treatment per case. At endpoint, cells in six-well plates were rinsed two times with PBS and lysed with TRIzol® reagent. Triplicate wells were pooled and stored in screw cap tubes at −80°C until ready for RNA extraction. RNA extraction was carried out as above for qRT-PCR experiments. RNA quality was analyzed using the Experion™ System (BIO-RAD, Hercules, CA, USA) and the Experion™ RNA StdSens Analysis Kit. RNA was then labelled and hybridized to Affymetrix Genechip® PrimeView™ Human Gene Expression Arrays (Santa Clara, CA, USA) according to the manufacturer’s instructions.

### Statistical analysis

Individual experiments were repeated at least three times with cells derived from separate cases. Each condition was performed in triplicate and mean +/−standard deviation is presented from representative experiments. Statistical analysis was performed on replicates within experiments using one-way analysis of variance (ANOVA) and Dunnett’s multiple comparison tests for significance with GraphPad Prism® analysis software.

For the microarray analysis, PrimeView™ array data in CEL file format was read using the ‘affy’ package in the statistical language R and normalized using the robust multi-array (RMA) method. Quality assurance (QA) of the data showed that it was of good quality and free of obvious artefacts or outliers. The ‘limma’ package was used to compare differential expression between the control (vehicle-treated) and IFNγ/IL-1β-treated samples. Benjamini-Hochberg false-discovery rate control was used to adjust for multiple testing.

## Results

### Composition and inflammatory state of primary cells from adult human brain

**Figure 1:**
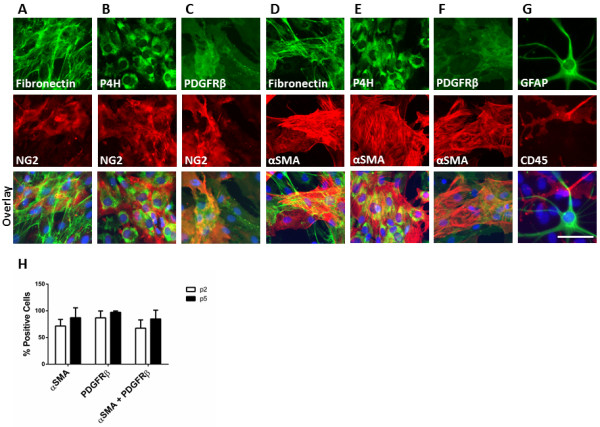
**Early passage mixed glial cultures are composed of astrocytes, microglia, fibroblasts and pericytes while later passages are primarily pericytes and/or fibroblasts.** Cells from at least three individual cases at passage 2 were stained by immunocytochemistry for the cell specific markers fibronectin **(A and D)** (green), prolyl-4 hydroxylase (P4H) **(B and E)** (green), and PDGFRβ **(C and F)** (green) and co-labelled with NG2 **(A-C)** (red) or αSMA **(D-F)** (red), or glial fibrillary acidic protein (GFAP) (green) and CD45 (red) **(G)**. Bottom images show overlay with Hoechst (blue) for all images. Scale bar = 100 μM. Percentages of αSMA, PDGFRβ or αSMA/PDGFRβ positive cells from passage 2 or 5 from three separate cases were quantified from triplicate wells, four images/well **(H)**. PDGFRβ, platelet-derived growth factor receptor-beta; αSMA, alpha smooth muscle actin.

**Figure 2:**
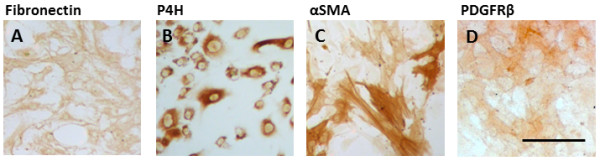
**Meningeal explant derived cells express fibroblast and pericyte markers.** Representative images from at least two cases of meningeal explant derived cells that were stained for **A)** fibronectin, **B)** P4H, **C)** αSMA and **D)** PDGFRβ. Scale bar = 100 μM. P4H, prolyl-4 hydroxylase; PDGFRβ, platelet-derived growth factor receptor-beta; αSMA, alpha smooth muscle actin.

Immunocytochemical analysis of dissociated cells from MTG after two weeks in culture revealed a mixed population of cells (referred to from now on as ‘mixed glial cultures’). Positive staining for fibronectin and prolyl-4 hydroxylase (P4H) (fibroblast markers), platelet-derived growth factor receptor-beta (PDGFR-β), NG2 and alpha smooth muscle actin (αSMA) (pericyte markers), CD45 (microglial cell marker), and glial fibrillary acidic protein (GFAP, astrocyte marker) was seen in early passages of dissociated cultures (Figure 1A-G). No endothelial cells were detected in any of our cultures using CD31 (data not shown). Composition of the mixed glial cultures varies from case to case, but we see 1-5% GFAP positive cells and 15% to 25% CD45 positive cells. In this initial culture, there are approximately 80% of cells that stain positively for both αSMA and PDGFRβ (Figure 1H). After subsequent passaging, CD45 and GFAP positive cells were lost and only the proliferating fibroblast-like and pericyte-like cells remained [26]. All cells in the later cultures are positive for fibronectin, and approximately 90% of cells are positive for both αSMA and PDGFRβ (Figure 1H). For purposes of simplicity, late passage cultures will be referred to as pericytes for the remainder of the manuscript. Considering the expression of fibroblast and pericyte specific markers it is likely that these dividing underlying cells derive from the meninges and brain microvasculature. To test this hypothesis, we resected the leptomeninges from the brain tissue and cultured them separately from the MTG cells. These explants were able to grow and divide in culture and generate new cells that migrate out of and away from the explant (unpublished observations). Positive staining for fibronectin, P4H, αSMA and PDGFR-β (Figure 2) was consistent with MTG cultures. Expression of both αSMA and PDGFR-β were confirmed in the MTG and explant cultures by western blot analysis showing one specific band at the expected molecular weight [see Additional file 3: Figure S1]. The NG2 antibody detected two bands which were expressed in both MTG and explant cultures [see Additional file 3: Figure S1].

Translocation of nuclear factor light chain enhancer of activated B cells (NFκB) is often used as a marker of a pro-inflammatory response. We examined NFκB p65 by immunocytochemistry in the mixed glial cultures treated with either vehicle, IFNγ, TNFα, IL-1β, or LPS to induce a pro-inflammatory response. Positive staining for NFκB was observed in the cytoplasm under control conditions after two hours of vehicle treatment. Treatment with TNFα, IL-1β or LPS induced translocation of NFκB to the nucleus in CD45, GFAP and αSMA positive cells [see Additional files 4, 5, 6: Figures S2, S3 and S4 respectively]. This result indicates that under our standard basal cell culture conditions adult human brain cells are in a ‘resting’ non-immunologically-activated state, making them a powerful system for studying human brain inflammation.

### Combination of IFNγ and IL-1β/TNFα synergistically induces IP-10 but not MCP-1 expression and secretion in mixed glial cultures

**Figure 3:**
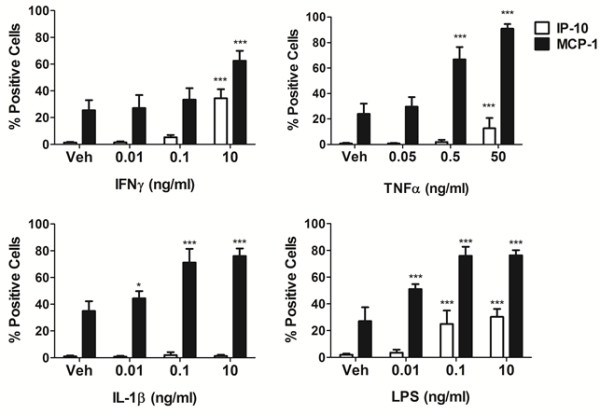
**Mixed glial cultures express chemokines IP-10 and MCP-1 when stimulated with pro-inflammatory molecules.** Quantification of cells immunocytochemically positive for IP-10 or MCP-1 in mixed glial cultures after 24 hours treatment with vehicle (0.1% BSA in PBS), interferon γ (IFNγ), tumor necrosis factor α (TNFα), interleukin 1β (IL-1β) or lipopolysaccharide (LPS). Values plotted are percentages of total cells as measured by Hoechst positive cells from triplicate wells, four images per well. Data are representative of results from three separate cases. *indicates significance (*P* <0.01), ***indicates significance (*P* <0.0001). IP-10, interferon inducible protein-10; MCP-1, monocyte chemotactic protein-1.

Previously, we have identified chemokines IP-10 and MCP-1 as being released into media from our mixed glial cultures as well as our pericyte cultures specifically in response to IFNγ treatment [27]. Confirmation of this result in mixed glial cultures by quantitative immunocytochemistry revealed an increase in IP-10 and MCP-1 expression in response to IFNγ treatment as demonstrated in Figure 3. We also tested the induction of IP-10 and MCP-1 protein expression in response to TNFα, IL-1β and LPS. We found a concentration dependent increase in both chemokines after IFNγ, TNFα and LPS treatment but only MCP-1 was induced by IL-1β (Figure 3). It is also important to note that basally IP-10 expression was not detectable, while MCP-1 expression was found in 25% to 40% of cells in untreated conditions.

**Figure 4:**
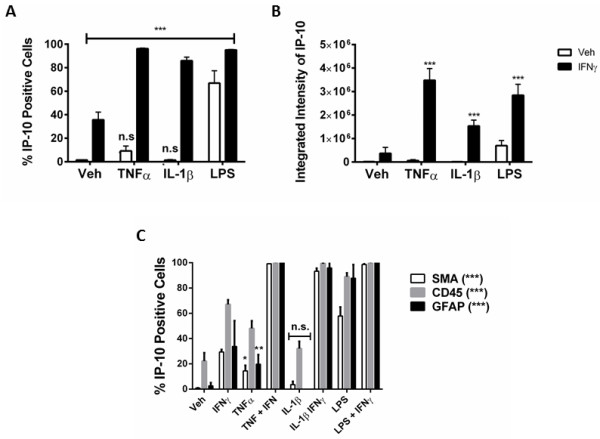
**IP-10 expression is synergistically increased in response to combination IFNγ and TNFα/IL-1β in human mixed glial cultures. A)**. Quantification of IP-10 positive cells in mixed glial cultures treated for 24 hours with vehicle (0.1% BSA in PBS), TNFα (50 ng/ml), IL-1β (10 ng/ml) or LPS (10 ng/ml) +/−IFNγ (10 ng/ml), from triplicate wells, at least four images per well. *indicates significance (*P* <0.01), ***indicates significance (*P* <0.0001). Data are from one representative experiment that was repeated in three separate cases. **B)** Quantification of the integrated intensity of IP-10 staining of cells treated in **(A)**, ***indicates significance (*P* <0.0001). **C)** Quantification of the percentage of αSMA, CD45 and GFAP positive cells that express IP-10 under conditions in **(A)**, *indicates significance (*P* <0.01), **(*P* <0.001), ***(*P* <0.0001). GFAP, glial fibrillary acidic protein; IP-10, interferon inducible protein-10; LPS, lipopolysaccharide; αSMA, alpha smooth muscle actin.

**Figure 5:**
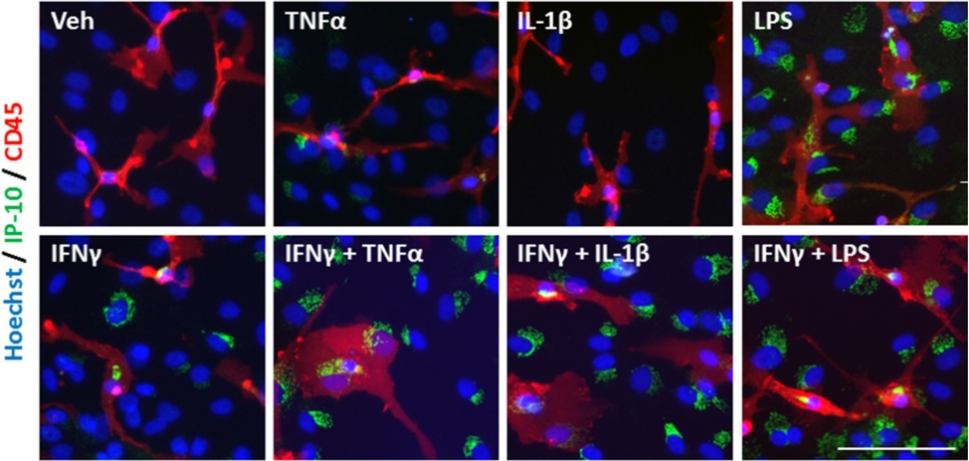
**IP-10 is induced in microglia in response to pro-inflammatory cues.** Representative images of cells in mixed glial cultures treated for 24 hours with vehicle (0.1% BSA in PBS), TNFα (50 ng/ml), IL-1β (10 ng/ml) or LPS (10 ng/ml) +/- IFNγ (10 ng/ml) labelled with IP-10 (green), CD45 (red) and Hoechst (blue), scale bar = 100 μm. IP-10, interferon inducible protein-10; LPS, lipopolysaccharide.

**Figure 6:**
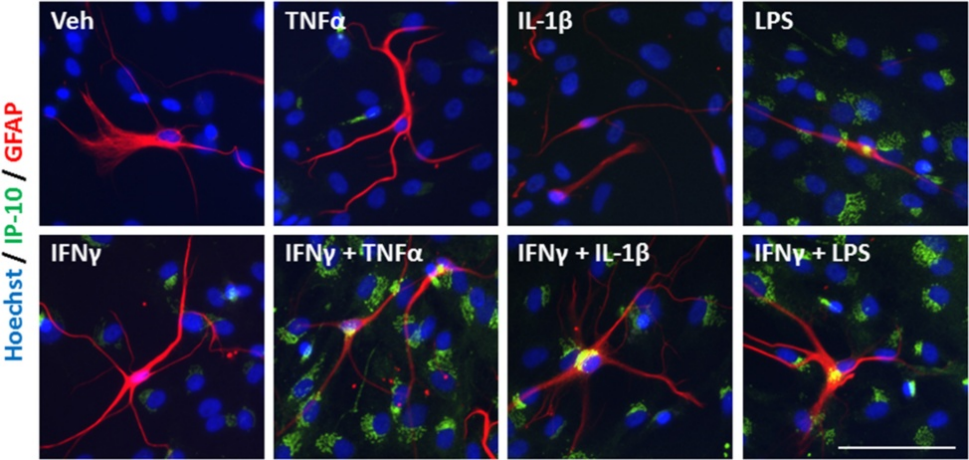
**IP-10 is induced in astrocytes in response to pro-inflammatory cues.** Representative images of cells in mixed glial cultures treated for 24 hours with vehicle (0.1% BSA in PBS), TNFα (50 ng/ml), IL-1β (10 ng/ml) or LPS (10 ng/ml) +/- IFNγ (10 ng/ml) labelled with IP-10 (green), GFAP (red) and Hoechst (blue), scale bar = 100 μm. GFAP, glial fibrillary acidic protein; IP-10, interferon inducible protein-10; LPS, lipopolysaccharide.

**Figure 7:**
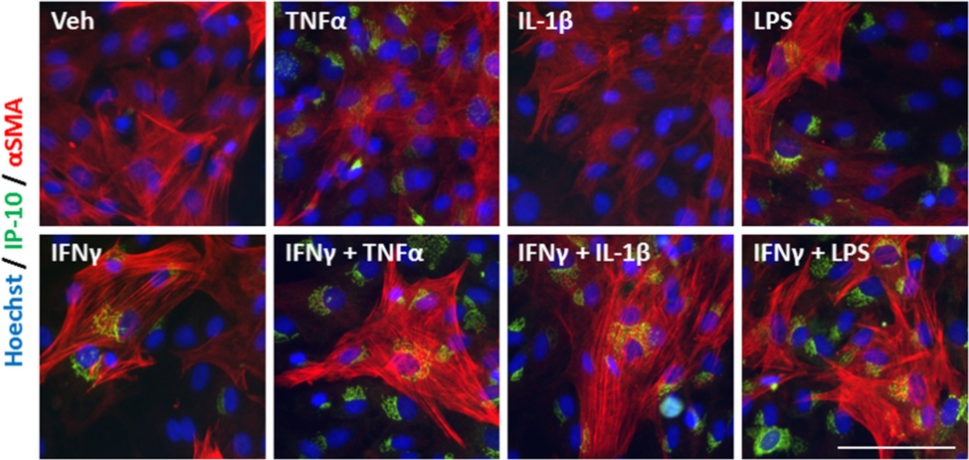
**IP-10 is induced in pericytes in response to pro-inflammatory cues.** Representative images of cells in mixed glial cultures treated for 24 hours with vehicle (0.1% BSA in PBS), TNFα (50 ng/ml), IL-1β (10 ng/ml) or LPS (10 ng/ml) +/- IFNγ (10 ng/ml) labelled with IP-10 (green), αSMA (red) and Hoechst (blue), scale bar = 100 μm. IP-10, interferon inducible protein-10; LPS, lipopolysaccharide; αSMA, alpha smooth muscle actin.

**Figure 8:**
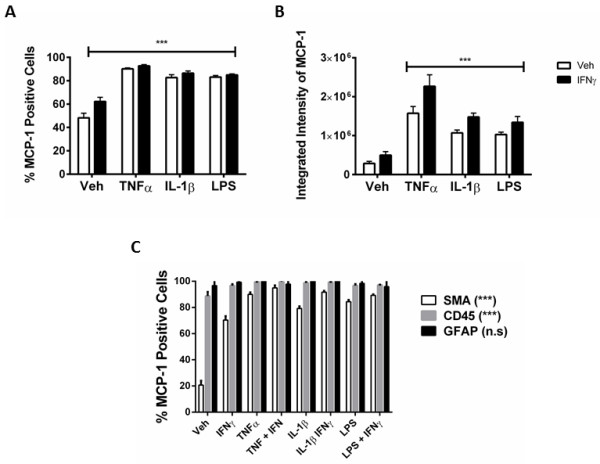
**MCP-1 expression is increased in response to IFNγ, TNF, IL-1β and LPS in human mixed glial cultures. A)** Quantification of MCP-1 positive cells in mixed glial cultures treated for 24 hours with vehicle (0.1% BSA in PBS), TNFα (50 ng/ml), IL-1β (10 ng/ml) or LPS (10 ng/ml) +/−IFNγ (10 ng/ml), from triplicate wells, at least four images per well. *indicates significance (*P* <0.01), ***indicates significance (*P* <0.0001). Data are from one representative experiment that was repeated in three separate cases. **B)** Quantification of the integrated intensity of MCP-1 staining of cells treated in **(A)**, ***indicates significance (P <0.0001). **C)** Quantification of the percentage of αSMA, CD45 and GFAP positive cells that express MCP-1 under conditions in **(A)**, *indicates significance (*P* <0.01), **(*P* <0.001), ***(*P* <0.0001). GFAP, glial fibrillary acidic protein; LPS, lipopolysaccharide; MCP-1, monocyte chemotactic protein-1; αSMA, alpha smooth muscle actin.

**Figure 9:**
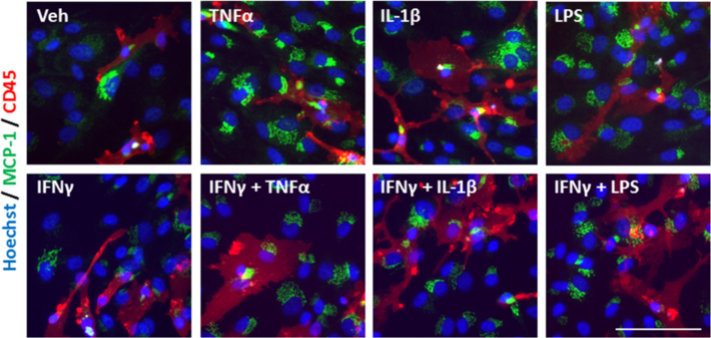
**MCP-1 is induced in microglia in response to pro-inflammatory cues.** Representative images of cells in mixed glial cultures treated for 24 hours with vehicle (0.1% BSA in PBS), TNFα (50 ng/ml), IL-1β (10 ng/ml) or LPS (10 ng/ml) +/- IFNγ (10 ng/ml) labelled with MCP-1 (green), CD45 (red) and Hoechst (blue), scale bar = 100 μm. LPS, lipopolysaccharide; MCP-1, monocyte chemotactic protein-1.

**Figure 10:**
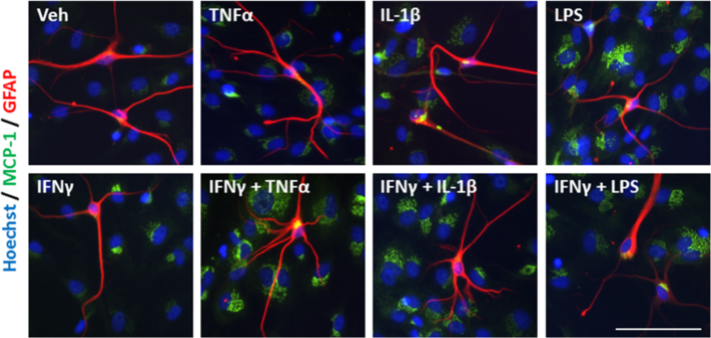
**MCP-1 is induced in astrocytes in response to pro-inflammatory cues.** Representative images of cells in mixed glial cultures treated for 24 hours with vehicle (0.1% BSA in PBS), TNFα (50 ng/ml), IL-1β (10 ng/ml) or LPS (10 ng/ml) +/- IFNγ (10 ng/ml) labelled with MCP-1 (green), GFAP (red) and Hoechst (blue), scale bar = 100 μm. GFAP, glial fibrillary acidic protein; LPS, lipopolysaccharide; MCP-1, monocyte chemotactic protein-1.

**Figure 11:**
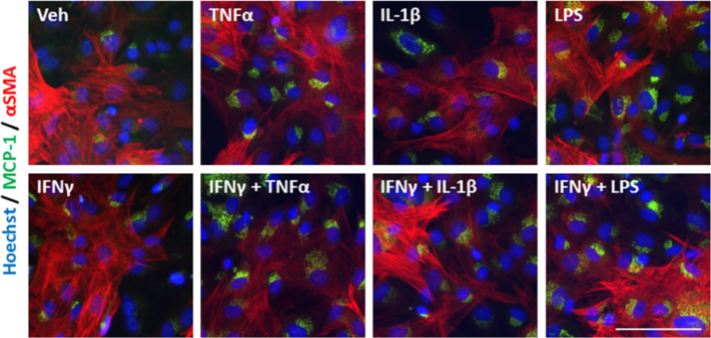
**MCP-1 is induced in pericytes in response to pro-inflammatory cues.** Representative images of cells in mixed glial cultures treated for 24 hours with vehicle (0.1% BSA in PBS), TNFα (50 ng/ml), IL-1β (10 ng/ml) or LPS (10 ng/ml) +/- IFNγ (10 ng/ml) labelled with MCP-1 (green), αSMA (red) and Hoechst (blue), scale bar = 100 μm. LPS, lipopolysaccharide; MCP-1, monocyte chemotactic protein-1; αSMA, alpha smooth muscle actin.

An inflammatory condition will rarely constitute the upregulation of a single cytokine, but many cytokines, chemokines and other inflammatory molecules [28,29]. We, therefore, examined the response of our mixed glial cultures to a combination of IFNγ and TNFα, IL-1β, or LPS treatment. Immunocytochemical examination of IP-10 in the mixed glial cultures revealed a synergistic increase in the number of cells expressing IP-10 when IFNγ was combined with either TNFα or IL-1β treatment (Figure 4A). Interestingly, although the combination of IFNγ and LPS increased the number of cells expressing IP-10 by only 20% above LPS alone, there was a further four-fold increase in the *intensity* of IP-10 staining by the combination, indicating a strong synergistic increase in overall protein expression per cell (Figure 4B). When we co-labelled with cell specific markers for microglia (CD45), astrocytes (GFAP) or pericytes (αSMA) we could also detect an increase in expression of IP-10 in each cell type, although the effect of combining LPS and IFNγ seems to be pericyte specific (Figures 4C, 5, 6, and 7). There was no significant change in the total number of astrocytes or microglia in response to any of the treatments (data not shown). MCP-1 expression was also increased in mixed glial cultures in response to pro-inflammatory stimuli; however, this also appears to be pericyte specific as basal expression of MCP-1 is detected in 90% to 100% of all microglia and astrocytes in these cultures under basal conditions (Figures 8, 9, 10 and 11). Moreover, the synergy seen with IP-10 expression by combining treatments is not present in the MCP-1 response.

### Brain pericytes respond to inflammatory cues by activating pro-inflammatory pathways in the absence of microglia and astrocytes

**Figure 12:**
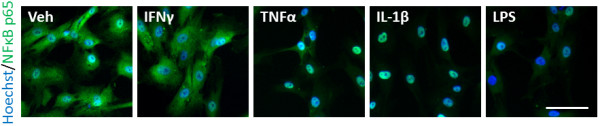
**Pericytes respond to inflammatory cues by nuclear translocation of NFκB.** Immunocytochemistry of NFκB p65 staining in primary human brain pericytes treated for two hours with vehicle (0.1% BSA in PBS), IFNγ (10 ng/ml), TNFα (50 ng/ml), IL-1β (10 ng/ml), or LPS (10 ng/ml). (NFκB p65 (green), Hoechst (blue)). Scale bar = 100 μm. Images are from one representative experiment that has been repeated in three separate cases. LPS, lipopolysaccharide; NFκB, nuclear factor light chain enhancer of activated B cells.

Although pericytes expressed IP-10 and MCP-1 in response to immune activators the contribution of co-cultured microglia and/or astrocytes to this response was not clear. Therefore, we tested late passage cultures in which microglia and astrocytes were absent. Treatment of pericytes with TNFα, IL-1β and LPS, but not IFNγ, showed clear nuclear translocation of NFκB p65 by immunocytochemistry after two hours (Figure 12).

Previous studies have shown the brain meninges to play a vital role in communication of inflammatory responses across the BBB [30-33]. We investigated the ability of meningeal explant cultures to respond to inflammatory stimuli. LPS and IL-1β but not IFNγ treatment induced NFκB translocation [see Additional file 7: Figure S5], which is consistent with our experiments in mixed glial and pericyte cultures. Furthermore, treatment with IFNγ induced IP-10 expression with LPS and IL-1β producing little or no effect, while MCP-1 expression was induced by all three treatments, IFNγ, IL-1β and LPS in our explant cultures [see Additional file 7: Figure S5].

**Figure 13:**
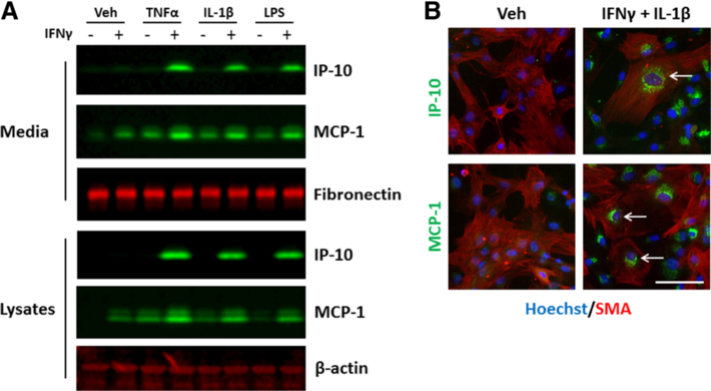
**IP-10 and MCP-1 are expressed and secreted by primary human brain pericytes. A)** Western blot analysis of media and lysates from brain pericytes treated with vehicle (0.1% BSA in PBS), TNFα (50 ng/ml), IL-1β (10 ng/ml) or LPS (10 ng/ml) +/−IFNγ (10 ng/ml) for 24 hours. Images are representative of experiments repeated in three separate cases. **B)** Immunocytochemical analysis of IP-10 and MCP-1 (both green) induction by IFNγ and IL-1β (both 10 ng/ml) treatment in αSMA (red) positive pericytes with Hoechst (blue) overlay. Scale bar = 100 μm. Images are representative of experiments repeated in three separate cases. IP-10, interferon inducible protein-10; LPS, lipopolysaccharide; MCP-1, monocyte chemotactic protein-1; αSMA, alpha smooth muscle actin.

Pericyte cultures responded similarly to the cytokine treatment in the absence of microglia or astrocytes. We examined IP-10 and MCP-1 expression by western blot of cell lysates and conditioned media and could see the same synergy of IP-10 induction in response to IFNγ + TNFα, or IFNγ + IL-1β compared to the cytokines alone (Figure 13A). It is noteworthy to mention that we did not see similar induction of IP-10 in response to LPS in pericytes alone compared to pericytes in mixed glial cultures. However, despite little to no induction of IP-10 by either IFN or LPS alone, we saw a significant increase in IP-10 protein expression and secretion in response to IFN and LPS together. This indicated that the concentration of LPS used was not saturating the system and indeed was within the range published in several other studies [34-37]. Consistent with previous results we see that basally MCP-1 is expressed at a higher level than IP-10. MCP-1 is also released into media under basal conditions and this is increased in response to IFNγ, TNFα, IL-1β and LPS and with combination treatment. To confirm the cell types responsible for chemokine release we performed immunocytochemistry on pericyte cultures treated with IFNγ and IL-1β. Our data showed that cells positive for MCP-1 and IP-10 in response to IFNγ and IL-1β are also positive for the pericyte marker αSMA (Figure 13B).

**Figure 14:**
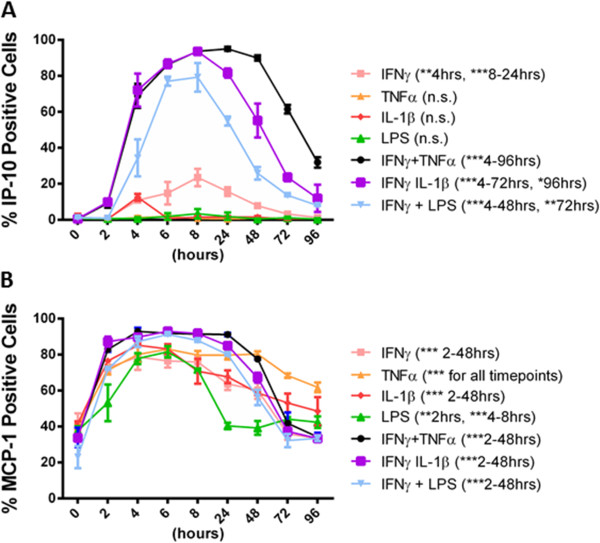
**Time-dependent expression of IP-10 and MCP-1 in response to pro-inflammatory cytokines in adult human brain pericytes. A)** Pericyte cultures treated for 0 to 96 hours with TNFα (50 ng/ml), IL-1β (10 ng/ml) or LPS (10 ng/ml) +/−IFNγ (10 ng/ml), were stained by immunocytochemistry for IP-10, and Hoechst and quantified by Hoechst positive cells from triplicate wells, four images per well. *indicates significance (*P* <0.01), ***indicates significance (*P* <0.0001). Data are from one representative experiment that was repeated in two separate cases. **B)** Pericyte cultures treated as in **(A)** were stained by immunocytochemistry for MCP-1 and quantified as above. IP-10, interferon inducible protein-10; LPS, lipopolysaccharide; MCP-1, monocyte chemotactic protein-1.

A time-dependent increase in both MCP-1 and IP-10 secretion by pericytes was seen by western blot analysis in response to IFNγ + TNFα, IL-1β or LPS treatment [see Additional file 8: Figure S6], which is consistent with immunocytochemical data (Figure 14A and B). IP-10 protein expression was induced in a time-dependent manner in pericytes by IFNγ and combined treatments, while MCP-1 was induced by all treatments. Accompanying the increase in protein expression was a sustained increase in secretion lasting at least 72 hours for IP-10 and 96 hours for MCP-1. We also examined the time-dependent regulation of IP-10 and MCP-1 in our pericyte culture system at the mRNA level by qRT-PCR [see Additional file 8: Figure S6]. Our results have shown that IP-10 and MCP-1 mRNA were present at extremely low levels, if at all, under basal conditions. However, mRNA expression was quickly induced by treatment with the inflammatory stimuli IFNγ and IL-1β. IP-10 mRNA was increased at one hour by almost 1,000-fold compared to vehicle control, with a maximum between 8 and 24 hours. MCP-1 was induced to a lesser extent, but by no means trivial at more than 10-fold after only 30 minutes treatment.

### Genome wide expression changes of immune pathways in human brain pericyte cultures

Since our qRT-PCR data revealed that treatment with the pro-inflammatory cytokines IFNγ + IL-1β could induce gene expression of the chemokines IP-10 and MCP-1, we sought to identify other regulatory mechanisms at the level of transcription in our pericyte cultures. The purpose of this experiment was to determine the initial changes in gene expression of pericytes in the absence of microglia or astrocytes. Cells from five separate cases were obtained with an age range of 45 +/−7 years, all male. Cells were treated with vehicle or IFNγ + IL-1β for 24 hours, a combination of cytokines modelling pathological events [38]. RNA was extracted and submitted for microarray analysis using Affymetrix Primeview arrays.

Primary analysis of microarray data confirmed the increased expression of both IP-10 and MCP-1 in response to IFNγ and IL-1β treatment. We also observed a significant increase in a plethora of genes involved in the immune response including interleukins (IL-6, IL-8, IL-32), chemokines (CXCL9, CXCL10, CXCL11, CCL2, CCL8), intracellular adhesion molecules (ICAM, VCAM), superoxide dismutase (SOD2), and apolipoprotein L (APOL1, APOL2, APOL3, APOL4, APOL6) by initial microarray analysis and by plotting raw data for individual genes [see Additional file 9: Figure S7]. Pathway analysis using the Gather online tool (http://gather.genome.duke.edu/) revealed enrichment for genes involved in immune response, complement activation, response to stress and chemotaxis (Table 1). Interestingly, and reassuringly, all five cases showed a very similar gene expression profile under control and induced conditions.

**Table 1:** **Gene ontology analysis of microarray data for pericytes treated with IFN**γ **and IL-1**β **for 24 hours**

**Annotation**	**Total genes with ann**	**Genome (with Ann)**	**Genes**
**GO:0006952**[5]**: defense response**	52	786	*APOL2 APOL3 C1R C1S C3 C4A C4B CCL2 CCL8 CD40 CD74 CTSS CXCL1 CXCL10 CXCL11 CXCL12 CXCL2 CXCL9 G1P2 GBP1 GBP2 GBP5 HLA-DMA HLA-E IFI30 IFI35 IFIT3 IL15 IL18BP IL32 IL6 IL8 IRF1 IRF8 MX1 NMI OAS2 OAS3 PSMB10 PSMB8 PSMB9 RIPK2 SAMHD1 SERPING1 SOD2 TAP1 TAP2 TAPBP TLR3 TNFRSF14 TNFSF13B UBD*
**GO:0009613**[5]**: response to pest, pathogen or parasite**	29	442	*APOL2 APOL3 C1R C1S C3 C4A C4B CCL2 CCL8 CD40 CD74 CXCL1 CXCL10 CXCL11 CXCL12 CXCL2 CXCL9 HLA-DMA IFI35 IL18BP IL6 IL8 NMI PSMB10 RIPK2 SERPING1 SOD2 TLR3 UBD*
**GO:0043207**[5]**: response to external biotic stimulus**	29	473	*APOL2 APOL3 C1R C1S C3 C4A C4B CCL2 CCL8 CD40 CD74 CXCL1 CXCL10 CXCL11 CXCL12 CXCL2 CXCL9 HLA-DMA IFI35 IL18BP IL6 IL8 NMI PSMB10 RIPK2 SERPING1 SOD2 TLR3 UBD*
**GO:0006954**[5]**: inflammatory response**	18	178	*APOL3 C3 C4A C4B CCL2 CCL8 CD40 CD74 CXCL1 CXCL10 CXCL11 CXCL12 CXCL2 CXCL9 IL8 NMI RIPK2 TLR3*
**GO:0009611**[5]**: response to wounding**	20	266	*APOL3 C3 C4A C4B CCL2 CCL8 CD40 CD74 CXCL1 CXCL10 CXCL11 CXCL12 CXCL2 CXCL9 IL18BP IL8 NMI RIPK2 SOD2 TLR3*
**GO:0006959**[5]**: humoral immune response**	12	151	*C1R C1S C3 C4A C4B CCL2 CD40 CD74 IL6 PSMB10 SERPING1 UBD*
**GO:0006935**[6]**: chemotaxis**	9	108	*CCL2 CCL8 CXCL1 CXCL10 CXCL11 CXCL12 CXCL2 CXCL9 IL8*
**GO:0006956**[6]**: complement activation**	6	33	*C1R C1S C3 C4A C4B SERPING1*
**GO:0006958**[7]**: complement activation, classical pathway**	5	25	*C1R C1S C3 C4A SERPING1*
**GO:0016064**[6]**: humoral defense mechanism (sensu Vertebrata)**	8	111	*C1R C1S C3 C4A CD40 CD74 SERPING1 UBD*
**GO:0042157**[7]**: lipoprotein metabolism**	5	30	*APOL1 APOL2 APOL3 APOL4 APOL6*

Analysis of the top 200 gene hits including fold changes of ≥2 with *P*-value ≤1.0 x 10^6^ reveals enrichment for genes involved in inflammatory and related events.

Although the general response of the primary cells to IFNγ and IL-1β was to increase gene expression, there was also downregulation of several genes including tumor necrosis factor receptor super family member 10D (TNFRSF10D, involved in inhibition of apoptosis [39]) CXCL12 (expressed in embryonic and post-natal meninges and acts as a chemoattractant to embryonic neurons [40]) and CREB5.

Genes selected for validation had a fold change of >2 in microarray with a *P* value of ≤1.0 x 10^−6^. Genes showing significant changes in expression by microarray were selected based on their involvement in biological processes relevant to the inflammatory process. Our validation data confirmed the results obtained by qRT-PCR, upregulated genes by microarray were also increased by qRT-PCR analysis and genes downregulated by microarray were also decreased in qRT-PCR (Table 2, Additional file 9: Figure S7 and Additional file 10: Figure S8).

**Table 2:** Gene expression changes in microarray and qRT-PCR validation

**Genes upregulated**	**Gene names**	**Fold change microarray**	**Fold change qRT-PCR**
*HLA-DMA*	major histocompatibility complex, class II, DM alpha	3.70 +/- 0.33	9.86 +/- 3.72
*ATF3*	activating transcription factor 3	2.33 +/- 1.0	5.49 +/- 0.45
*ICAM1*	intercellular adhesion molecule 1	6.13 +/- 0.56	80.80 +/- 23.88
*CXCL11*	chemokine (C-X-C motif) ligand 11	9.40 +/- 0.65	246926 +/- 216491
*IL-6*	interleukin-6	5.82	117.69 +/- 17.29
*IL-8*	interleukin-8	7.5 +/- 0.63	193.19 +/- 62.31
*CD74*	CD74 molecule, major histocompatibility complex, class II invariant chain	3.47 +/- 1.22	160.96 +/- 142.58
*SOD2*	superoxide dismutase 2, mitochondrial	4.35 +/- 1.03	27.38 +/- 3.90
*APOL1*	apolipoprotein L, 1	4.19 +/- 0.27	25.56 +/- 7.87
*APOL2*	apolipoprotein L, 2	3.46	8.34 +/- 1.94
*RIPK2*	receptor-interacting serine-threonine kinase 2	2.04 +/- 0.25	3.16 +/- 0.24
*IRF1*	interferon regulatory factor 1	4.48 +/- 0.39	21.55 +/- 3.25
*PTGS2*	prostaglandin-endoperoxide synthase 2 (prostaglandin G/H synthase and cyclooxygenase)	3.28 +/- 0.82	8.49 +/- 1.09
***Genes downregulated***			
*CREB5*	cAMP responsive element binding protein 5	-1.80 +/- 0.83	0.1 +/- 0.03
*TNFRSF10D*	tumor necrosis factor receptor superfamily, member 10d, decoy with truncated death domain	-4.78	0.05 +/- 0.02
*CXCL12*	chemokine (C-X-C motif) ligand 12	-1.79 +/- 0.25	0.39 +/- 0.18

Fold changes from vehicle +/- SD in microarray experiment as well as qRT-PCR validation.

## Discussion

In this study we examine the effects of inflammatory stimuli on primary adult human brain derived cells. These mixed populations of cells stain positively for microglia, astrocyte, fibroblast and pericyte markers. Since microglia and astrocytes do not normally proliferate in culture, after passaging only pericyte and fibroblast cells remain [17,23,41]. We hypothesize that the pericyte cells originate from the microvasculature and meninges and, in fact, we see a consistency in cell specific markers and response to inflammatory cues in our dissociated and leptomeningeal explant cultures. Using a number of markers in this and previous studies (CD45, PU.1, GFAP), we are confident with the identification of microglia and astrocytes in our cultures [17,26,41].

More recently, using markers of pericytes (αSMA, PDGFRβ, NG2) and fibroblasts (P4H, fibronectin), we have been able to identify the proliferative population of cells from adult human brain cultures as a mix of brain pericytes and fibroblast-like cells. However, currently there is much debate in the literature about the *in vitro* identification of pericytes and, in particular, how they are distinguished (or not) from mesenchymal stem cells or fibroblasts, because there is no one specific marker for any of these cells [42,43]. The fact that fibronectin is detected in our cultures is in line with earlier studies as fibronectin has been shown to be expressed in pericyte cell lines [44]. Furthermore, αSMA is expressed in pericytes at different levels dependent on the state of differentiation and treatment conditions as well as location on capillaries [36,45-47] (and unpublished observations). PDGFRβ is a more widely used marker for pericytes; however, it is also expressed in other cell types such as fibroblasts and astrocytes and can be upregulated in response to stresses such as injury and inflammation [48-50]. In double-label studies, we found that 80% to 90% of the cells (depending upon passage number) expressed both αSMA and PDGFRβ. The other pericyte marker used here is NG2 which is a marker commonly used to identify pericytes; however, it is also present in meningeal fibroblasts and other precursor cell types [51,52]. Furthermore, we see a difference in NG2 band distribution by western blot when comparing pericyte cells to meningeal explant cells. This may be due to differential expression of NG2 isoforms in either cell type or due to phosphorylation events which play a role in polarity and migration [53,54]. For the above reasons, although we have operationally defined these cells as pericytes (based on αSMA, PDGFRβ and NG2 staining), it is possible that they represent a mixed cell population that may additionally contain mesenchymal stem cells and fibroblast-like cells. It is important to note that we do not see positive staining for endothelial cell markers and this may be due to the fact that we do not use any supplements specific for endothelial cell culture as in previous studies [55,56].

Under our standard basal cell culture conditions, the human brain-derived microglia, astrocytes and pericytes expressed only cytoplasmic NFκB staining suggesting that these cells under our normal culture conditions are in a non-immunologically-activated state, making them a powerful system for studying human brain inflammation *in vitro*. To activate these cells we use cytokines IFNγ, TNFα and IL-1β as well as the bacterial endotoxin LPS, which are commonly elevated in conditions of chronic peripheral and central inflammation [57-60].

A common indicator of an inflammatory response is translocation of the transcription factor NFκB to the nucleus; therefore, we have used this as an initial marker of a pro-inflammatory state [61,62]. We have shown that both the early and late cultures will respond to inflammatory cues by inducing nuclear translocation of NFκB from the cytoplasm. This has been corroborated in rodent astrocytes and microglia and more recently by Guijarro-Munoz and colleagues in human pericyte cells in response to LPS [37,63,64]. Interestingly, we did not see this with IFNγ treatment, which remains controversial in other studies [65,66]. Additionally, inhibition or blockade of the NFκB pathway has been shown to protect from LPS-induced neurotoxicity [67,68]. This uncovers potential targets for anti-inflammatory drug development, which is supported by *in vitro* evidence in human brain cells from our group and others [37].

We have also observed that pericytes upregulate protein expression of chemokines, specifically IP-10 and MCP-1 in response to various immune stimuli, and this is concentration and time-dependent. These results support previous studies using human and mouse brain pericytes showing immune activation with LPS [29,37]. Our studies build on and extend this previous work to show that a range of cytokines, in addition to LPS, can cause wide-spread pro-inflammatory immune activation of human brain pericytes.

Induced MCP-1 expression has been shown in T-cell infiltration areas and increases immune cell recruitment in animal models of central inflammation [69,70]. This may be in part due to the ability of MCP-1 to enhance permeability of BBB as shown in co-culture models of endothelial cells and astrocytes [71]. Increased expression of IP-10 and MCP-1 has also been implicated in MS, AD and HIV-related dementia and has been shown to induce neuronal toxicity in *in vitro* models [69,72-75]. The evidence is quite clear that these two chemokines play a role in inflammation and, possibly, neurodegeneration in the brain. Further studies examining the expression of IP-10 and MCP-1 in our human primary brain cultures will enable us to determine more precisely their role in human brain inflammation and disease.

Quantification of chemokine expression in the mixed cultures revealed a higher level of expression of IP-10 in microglia and MCP-1 in both microglia and astrocytes under basal and treated conditions compared to pericytes. Analysis of IP-10 and MCP-1 expression in the presence or absence of microglia and astrocytes has revealed similar responses by pericytes in both conditions. We see synergistic induction of IP-10 in response to a combination of IFNγ and TNFα/IL-1β or LPS by pericytes in both circumstances. However, while LPS alone induced pericyte MCP-1 expression in both conditions, IP-10 induction was only observed in early passage cultures containing microglia and astrocytes. This may be due to factors released specifically by the microglia and astrocytes themselves in response to LPS that cause IP-10 induction. Alternatively, it might be due to changes in pericytes with passaging. Further studies investigating early passage pericyte monocultures are necessary to test these possibilities.

Anatomically, pericytes surround endothelial cells and are in contact with astrocytes, neurons and other glial cells. Together these cells comprise the neurovascular unit [76,77]. We have shown that αSMA positive pericytes secrete chemokines and cytokines into media in response to pro-inflammatory cues in the absence of microglia or astrocytes. Other groups have also shown that pericytes can secrete factors that are essential for proper BBB functioning and can affect gene and protein expression in surrounding endothelial cells [78,79]. Co-culture studies of brain pericytes and endothelial cells indicate an important role in maintenance of hemostasis as well as BBB permeability to pathogens, such as HIV-1, in human and mouse cells, respectively [80,81]. Cytokine-induced release of chemokines and other inflammatory mediators by pericytes may be able to act in an autocrine and paracrine fashion to damage cells of the neurovascular unit and disrupt the BBB. An example of this has been shown by disruption of endothelial junctions by MCP-1, which impairs proper BBB maintenance and functioning [71,82]. Although it has been discovered that pericytes have differential roles in fetal or young compared to aged animals, these cells are essential for proper brain functioning. Loss of pericytes in mouse brain microvasculature leads to BBB permeability and neuronal cell death that worsens with age [78,83]. This is significant because BBB disruption is a feature not only of central inflammation but appears to be an early dysfunction associated with a number of neurodegenerative disorders such as epilepsy, motor neuron disease and Alzheimer’s disease (reviewed [84-86]).

It is clear that pericytes play a key role in the neurovascular unit of the BBB and when performing appropriately are vital for brain cell survival. However, in circumstances such as chronic inflammation, these cells can be harmful for BBB integrity and brain function. To understand better the role of pericytes in inflammation-mediated pathologies we investigated global gene expression changes in response to pro-inflammatory cytokines in primary adult human brain pericytes. Gene hits obtained from our microarray study show increases in IFNγ-regulated genes, interleukins (IL-6, IL-8, IL-15 and IL-32) and interleukin associated receptors, which have known roles in inflammatory processes in a number of different cell types and tissues [87-92]. We also see an increase in activating transcription factor 3 (ATF3) gene expression which is activated in response to stress and may be acting as a negative feedback mechanism to inhibit IL-6 expression [93,94]. Indoleamine 2,3-dioxygenase (IDO-1), which regulates tryptophan metabolism, was also strongly induced in the array study. This enzyme has been implicated in immune suppression and levels of IDO-1 are elevated in the neurofibrillary tangles of AD [95]. Chemokines IP-10 (CXCL10), CXCL11 and CXCL9 showed the largest increase by microarray, and both IP-10 and CXCL11 were validated by qRT-PCR. The role of IP-10 has been discussed; however, CXCL9 and CXCL11 are also important in peripherally mediated-CNS inflammation [6,96-98]. Interestingly CXCL9, −10 and −11 inhibit endothelial and perivascular cell growth which may contribute to BBB breakdown [99-101]. As previously mentioned, MCP-1 expression was also increased in our microarray experiment and reduces BBB integrity in *in vitro* and *in vivo* studies [71]. Rat pericytes have shown the ability to express major histocompatibility complex (MHC) class II antigen presenting molecules (such as HLA, major histocompatibility complex, class II DR alpha (HLA)) as well as intracellular adhesion molecules (ICAM) in response to IFNγ treatment, which is consistent with our results of upregulated HLA and ICAM expression in response to cytokine treatment [102]. Particularly, ICAM may be involved in regulating immune responses by enhancing leukocyte interactions [10]. We also observe an increase in the expression of CD74, a membrane protein that is found surrounding neurofibrillary tangles in AD [103]. Pro-apoptotic, lipid binding proteins apolipoprotein (APOL) -1, −2, −3, −4, and −6 were all increased in response to IFNγ and IL-1β treatment which is consistent with previous work in mouse embryonic fibroblast cells [104]. Other gene hits that have been verified by qRT-PCR include interferon regulatory factor-1 (IRF1), superoxide dismutase-2 (SOD2) and prostaglandin-endoperoxide synthase-2 (PTGS2) or COX-2. IRF1 mediates mouse microglial cell death in response to IFNγ and LPS [105]. COX-2 is often used as a marker for neuronal inflammation and has shown some beneficial effects in decreasing the risk of inflammatory mediated AD [106,107]. SOD2 dysregulation contributes to AD pathology in transgenic mice and neuronal cell death after ischemia [107-109]. We also see a downregulation of several genes, CXCL12, TNFRSF10D and CREB5, which were confirmed by qRT-PCR validation. CXCL12 is involved in chemoattraction but also in neuromodulation, as it affects neuronal signaling and CREB activation, and is involved in neurotransmitter release and modulation of voltage gated channels [110,111]. Several studies have indicated a protective role for CXCL12 in stroke-induced brain injury [112,113]. Although under our conditions and timepoints there is no evidence of cell death of pericyte cells, we do see an increase in TNFSF10/TRAIL and a decrease in TNFRSF10D expression by microarray, two genes that activate and inhibit cell death mechanisms, respectively, in response to IFNγ [114,115]. Further experiments will help to determine whether a pro-inflammatory environment will induce cell death of human brain pericytes.

In our study, we have shown that principal immune cells, such as microglia and astrocytes, are not alone in the inflammatory response as it pertains specifically to the human brain. Adult human meningeal and microvascular derived fibroblast and pericyte cells demonstrate the ability to respond to pro-inflammatory cues and activate a variety of signaling cascades to transmit a pro-inflammatory response to principal immune and other brain cells. However, caution must be used when interpreting data of this kind from *in vitro* cultures as culture conditions (media type, supplements added and so on) can greatly affect the physiology and biology of the cell system examined. Any information obtained from studies such as these should be verified in human tissue where possible.

## Conclusions

Our studies show that human brain pericytes as well as microglia and astrocytes can generate a pro-inflammatory response after immune challenge *in vitro*. Drugs that reduce pericyte-mediated inflammation might be beneficial in restoring BBB function and reducing brain inflammation and injury in brain disorders such as epilepsy, stroke, motor neuron disease, and Alzheimer’s disease, as well as many other neurodegenerative disorders.

## Abbreviations

AD: Alzheimer’s disease; APP: amyloid precursor protein; BBB: blood brain barrier; BSA: bovine serum albumin; CSF: cerebrospinal fluid; CXCR3: chemokine CXC motif receptor 3; CCR2: chemokine cc motif receptor 2; CNS: central nervous system; CREB: cyclic-AMP response element binding protein; (D)MEM: (Dulbecco’s) modified Eagle’s medium; FBS: fetal bovine serum; GFAP: glial fibrillary acidic protein; ICAM: intercellular adhesion molecule; IDO-1: indoleamine 2,3-dioxygenase; HLA: major histocompatibility complex, class II DR alpha; IFNγ: interferon-gamma; IP-10: interferon inducible protein-10; IL-1β: interleukin-1β; IκB: inhibitor of κB; LPS: lipopolysaccharide; IRF1: interferon regulatory factor-1; MCP-1: monocyte chemotactic protein-1; MHC: major histocompatibility complex; MS: multiple sclerosis; MTG: middle temporal gyrus; NFκB: nuclear factor light chain enhancer of activated B cells; PBS-T: phosphate buffered saline with Tween; PNS: peripheral nervous system; PET: positron emission tomography; PDGFRβ: platelet-derived growth factor receptor-β; P4H: prolyl-4 hydroxylase; PTGS2: prostaglandin-endoperoxide synthase-2; qRT-PCR: quantitative reverse transcriptase-polymerase chain reaction; RMA: robust multi-array; SOD2: superoxide dismutase-2; SMA: smooth muscle actin; TNFα: tumor necrosis factor–α; TNFRSF10D: tumor necrosis factor receptor superfamily member 10D; TBS-T: tris buffered saline with Tween; VCAM: vascular cell adhesion molecule.

## Competing interests

The authors declare that they have no competing interests.

## Authors’ contributions

DJ and MD designed the experiments. DJ carried out the experiments with mixed glial and pericyte cultures as well as the microarray experiment. JR completed qRT-PCR confirmation of microarray hits including primer design and standard curves for all primers used. SF completed the leptomeningeal explant experiments and characterization. DH performed statistical analysis for the microarray study as well as general revisions of the manuscript. RLO, PSB, EWM, RLMF and MD contributed materials and edited and reviewed the manuscript. All authors read and approved the final manuscript.

## Supplementary Material

Additional file 1: Table S1 Antibodies used in this study. Click here for file

Additional file 2: Table S2 Primers used for validation of microarray genes hits by qRT-PCR. Click here for file

Additional file 3: Figure S1 Western blot analysis of pericyte culture extracts (P) and explant culture extracts (E) in untreated conditions confirming specificity of antibodies used for immunocytochemistry of αSMA, PDGFR-β and NG2. Click here for file

Additional file 4: Figure S2 NFκB p65 translocates to the nucleus after treatment with TNFα, IL-1β, and LPS in microglia from mixed glial cultures. Mixed glial cultures treated with vehicle (0.1% BSA in PBS), IFNγ (10 ng/ml), TNFα (50 ng/ml), IL-1β (10 ng/ml) or LPS for two hours then stained by immunocytochemistry for NFκB p65 (green), CD45 (red), and Hoechst (blue). Scale bar =100 μm. Images are representative of experiments done in triplicate, repeated in two separate cases. Click here for file

Additional file 5: Figure S3 NFκB p65 translocates to the nucleus after treatment with TNFα, IL-1β, and LPS in astrocytes from mixed glial cultures. Mixed glial cultures treated with vehicle (0.1% BSA in PBS), IFNγ (10 ng/ml), TNFα (50 ng/ml), IL-1β (10 ng/ml) or LPS for two hours then stained by immunocytochemistry for NFκB p65 (green), GFAP (red) and Hoechst (blue). Scale bar =100 μm. Images are representative of experiments done in triplicate, repeated in two separate cases. Click here for file

Additional file 6: Figure S4 NFκB p65 translocates to the nucleus after treatment with TNFα, IL-1β, and LPS in pericytes from mixed glial cultures. Mixed glial cultures treated with vehicle (0.1% BSA in PBS), IFNγ (10 ng/ml), TNFα (50 ng/ml), IL-1β (10 ng/ml) or LPS for two hours then stained by immunocytochemistry for NFκB p65 (green) and αSMA (red) and Hoechst (blue). Scale bar =100 μm. Images are representative of experiments done in triplicate, repeated in two separate cases. Click here for file

Additional file 7: Figure S5 Leptomeningeal explant cultures express IP-10 and MCP-1 and activate NFκB nuclear translocation in response to pro-inflammatory stimuli. **A**. Quantification of NFκB translocation in leptomeningeal explant cultures after two hours treatment with LPS (10 ng/ml), IL-1β (10 ng/ml), or IFNγ (10 ng/ml) labelled with NFκB p65. This was analyzed using Metamorph Image Analysis software (nuclear translocation assay). **B**. Quantification of IP-10 staining intensity in cells immunocytochemically positive for IP-10 expression after 24 hours treatment with LPS (10 ng/ml), IL-1β (10 ng/ml), or IFNγ (10 ng/ml). **C**. Quantification of MCP-1 staining intensity in cells immunocytochemically positive for MCP-1 expression after 24 hours treatment LPS (10 ng/ml), IL-1β (10 ng/ml), or IFNγ (10 ng/ml). Analysis is representative of experiments repeated in at least three cases. Click here for file

Additional file 8: Figure S6 IP-10 and MCP-1 mRNA expression and protein secretion increases in a time-dependent manner in human brain pericyte cells. **A**. Western blot analysis of IP-10 and MCP-1 secretion in conditioned media from late passage primary brain cells in response to combination treatment of TNFα (50 ng/ml), IL-1β (10 ng/ml) and LPS (10 ng/ml) with IFNγ (10 ng/ml). Western blot is representative of experiment repeated in two separate cases. **B**. qRT-PCR time-course analysis measuring IP-10 and MCP-1 gene expression in primary brain cells treated with IFNγ (10 ng/ml) and IL-1β (10 ng/ml). Data are representative of experiment repeated in two separate cases. Click here for file

Additional file 9: Figure S7 Analysis of raw data from microarray experiment of genes chosen for validation by qRT-PCR reveals significant changes in gene expression. Each graph represents raw data from microarray experiments for the genes selected for confirmation by qRT-PCR. Each point is the expression of that gene by each patient sample in the designated condition (Vehicle (Veh) or IFNγ + IL-1β treated). Click here for file

Additional file 10: Figure S8 Analysis of ΔCt values from qRT-PCR validation experiments reveals significant changes in gene expression across passages. Each graph represents mean ΔCt values for vehicle or IFNγ + IL-1β treated cultures. This experiment was repeated three times in one case over three successive passages. Click here for file
