# Erratum to: A role for human brain pericytes in neuroinflammation

**DOI:** 10.1186/s12974-015-0430-5

**Published:** 2015-11-19

**Authors:** Deidre Jansson, Justin Rustenhoven, Sheryl Feng, Daniel Hurley, Robyn L. Oldfield, Peter S. Bergin, Edward W. Mee, Richard L. M. Faull, Mike Dragunow

**Affiliations:** Department of Pharmacology and Clinical Pharmacology, The University of Auckland, 85 Park Road, Auckland, 1023 New Zealand; Gravida National Centre for Growth and Development, The University of Auckland, Bldg 505, 85 Park Road, Auckland, 1023 New Zealand; Department of Anatomy with Radiology, The University of Auckland, Bldg 505, 85 Park Road, Auckland, 1023 New Zealand; Centre for Brain Research, The University of Auckland, Bldg 503, 85 Park Road, Auckland, 1023 New Zealand; Department of Molecular Medicine and Pathology, The University of Auckland, Bldg 504, 85 Park Road, Auckland, 1023 New Zealand; LabPLUS, Auckland City Hospital, Bldg 31, Gate 4 Grafton Road, Auckland, 1148 New Zealand; Auckland City Hospital, 2 Park Rd, Auckland, 1010 New Zealand

With regard to our recent article [1], please note that some explant cultures were also generated from autopsy human brain.
