# Supplementary material for: A role for human brain pericytes in neuroinflammation
Source: J Neuroinflammation. 2014 Jun 11;11:104. doi: 10.1186/1742-2094-11-104 (PMC4105169; doi:10.1186/1742-2094-11-104)
Supplement: Additional file 1: Table S1 — Antibodies used in this study. [file 1742-2094-11-104-S1.docx]

**Supplementary Table 1: Antibodies used in this study.**

| **Antibodies** | **Company** | **Catalogue** | **ICC** | **WB** |
| --- | --- | --- | --- | --- |
| αSMA mouse monoclonal | Dako | IS611 | ½ | 1/5 |
| β-actin mouse monoclonal | Abcam | ab6276 |  | 1/2000 |
| CD45 mouse monoclonal | Abcam | ab8216 | 1/500 |  |
| Fibronectin rabbit polyclonal | Dako | A0245 | 1/1.0x10^6^ | 1/1.0x10^6^ |
| GFAP mouse monoclonal | Cell Signaling | mAb3670 | 1/1.0x10^4^ |  |
| GFAP rabbit polyclonal | Dako | Z0334 | 1/1.0x10^4^ |  |
| IP-10 rabbit polyclonal | Abcam | ab9807 | 1/500 | 1/1000 |
| MCP-1 rabbit polyclonal | Abcam | ab9669 | 1/500 | 1/1000 |
| NFκB p65 (C-20) rabbit polyclonal | Santa Cruz | sc-372 | 1/500 |  |
| NG2 mouse monoclonal | Santa Cruz | sc-53389 | 1/500 | 1/1000 |
| P4H rabbit polyclonal | Sigma | HPA0075991 | 1/500 |  |
| PDGFRβ rabbit monoclonal | Cell Signaling | mAb3169 | 1/500 | 1/1000 |
| PU.1 rabbit monoclonal | Cell Signaling | mAb2258 | 1/500 |  |
| Alexa Fluor® 488 Goat anti-rabbit IgG (H+L) | Life Technologies | A11034 | 1/500 |  |
| Alexa Fluor® 594 goat anti-mouse IgG (H+L) | Life Technologies | A11032 | 1/500 |  |
| Goat anti-mouse IRDye-680LT | LiCOR | 926-68020 |  | 1/ 2.0x10^4^ |
| Goat anti-rabbit IRDye-800CW | LiCOR | 926-32211 |  | 1/ 2.0x10^4^ |
| Goat anti-rabbit IgG (whole molecule) biotin conjugate | Sigma | B7389 | 1/500 |  |
| Goat anti-mouse IgG (whole molecule) biotin conjugate | Sigma | B7264 | 1/500 |  |
| ECL Mouse IgG, HRP-linked whole Ab from sheep | GE Healthcare | NA931 |  | 1/2000 |
| ECL Rabbit IgG, HRP-linked whole Ab from donkey | GE Healthcare | NA934 |  | 1/2000 |

List of antibodies used as well as dilutions used for either immunocytochemistry (ICC) or western blot (WB).
