# Supplementary material for: A role for human brain pericytes in neuroinflammation
Source: J Neuroinflammation. 2014 Jun 11;11:104. doi: 10.1186/1742-2094-11-104 (PMC4105169; doi:10.1186/1742-2094-11-104)
Supplement: Additional file 2: Table S2 — Primers used for validation of microarray genes hits by qRT-PCR. [file 1742-2094-11-104-S2.docx]

**Supplementary Table 2: Primers used for validation of microarray genes hits by qRT-PCR.**

| Accession Number | Gene |  | Sequence | Amplicon Length | Primer Efficiency |
| --- | --- | --- | --- | --- | --- |
| NM_002046.4 | GAPDH (h) | Fw | CATGAGAAGTATGACAACAGCCT | 113 bp | 98.4% |
|  |  | Rv | AGTCCTTCCACGATACCAAAGT |  |  |
| NM_006120.3 | HLA-DMA (h) | Fw | ACGACGAGGACCAGCTTTTC | 81 bp | 99.2% |
|  |  | Rv | GAGCCCAGTCAGCAAATTCG |  |  |
| NM_001674.3 | ATF3 (h) | Fw | ACCCAAAACCCTGAAGCCAT | 73 bp | 94.8% |
|  |  | Rv | TGATGGTTCTCTGCTGCTGG |  |  |
| NM_000201.2 | ICAM1 (h) | Fw | GAACCAGAGCCAGGAGACAC | 84 bp | 100.9% |
|  |  | Rv | GAGACCTCTGGCTTCGTCAG |  |  |
| NM_005409.4 | CXCL11 (h) | Fw | GAGTGTGAAGGGCATGGCTAT | 70 bp | 97.5% |
|  |  | Rv | CATGGGGAAGCCTTGAACAAC |  |  |
| NM_000600.3 | IL-6 (h) | Fw | TTCGGTCCAGTTGCCTTCTC | 77 bp | 98.3% |
|  |  | Rv | TCTTCTCCTGGGGGTACTGG |  |  |
| [NM_000584.3](http://www.ncbi.nlm.nih.gov/entrez/viewer.fcgi?db=nucleotide&id=324073503) | IL-8 (h) | Fw | CAGAGACAGCAGAGCACACA | 70 bp | 102.0% |
|  |  | Rv | GTGAGATGGTTCCTTCCGGT |  |  |
| [NM_001025159.2](http://www.ncbi.nlm.nih.gov/entrez/viewer.fcgi?db=nucleotide&id=343403785) | CD74 (h) | Fw | GAGTCACTGGAACTGGAGGAC | 81 bp | 96.1% |
|  |  | Rv | CTGCTCTCACATGGGGACTG |  |  |
| [NM_000636.2](http://www.ncbi.nlm.nih.gov/entrez/viewer.fcgi?db=nucleotide&id=67782304) | SOD2 (h) | Fw | GCCCTGGAACCTCACATCAA | 79 bp | 100.6% |
|  |  | Rv | TCAGGTTGTTCACGTAGGCC |  |  |
| [NM_003661.3](http://www.ncbi.nlm.nih.gov/entrez/viewer.fcgi?db=nucleotide&id=211938437) | APOL1 (h) | Fw | ATGTGGCCCCTGTAAGCTTC | 79 bp | 103.3% |
|  |  | Rv | GCCCCCTCATGTAAGTGCTT |  |  |
| NM_030882.2 | APOL2 (h) | Fw | CCATGAACCCAGAGAGCAGT | 70 bp | 101.6% |
|  |  | Rv | TCTCTGCTCACTTGGTCCTG |  |  |
| [NM_003821.5](http://www.ncbi.nlm.nih.gov/entrez/viewer.fcgi?db=nucleotide&id=93141034) | RIPK2 (h) | Fw | CTGCCAGCTCCTCAAGACAA | 82 bp | 95.9% |
|  |  | Rv | TTCCAGGACAGTGATGCAGC |  |  |
| [NM_002198.2](http://www.ncbi.nlm.nih.gov/entrez/viewer.fcgi?db=nucleotide&id=196049386) | IRF1 (h) | Fw | TGCTTCCACCTCTCACCAAG | 84 bp | 96.8% |
|  |  | Rv | ACTTCCTCTTGGCCTTGCTC |  |  |
| [NM_000963.2](http://www.ncbi.nlm.nih.gov/entrez/viewer.fcgi?db=nucleotide&id=223941909) | PTGS2 (h) | Fw | AGGGTTGCTGGTGGTAGGAA | 76 bp | 97.7% |
|  |  | Rv | TCTGCCTGCTCTGGTCAATG |  |  |
| [NM_182898.2](http://www.ncbi.nlm.nih.gov/entrez/viewer.fcgi?db=nucleotide&id=59938769) | CREB5 (h) | Fw | ATCAATCATCACGGCCTGCA | 71 bp | 109.0% |
|  |  | Rv | GAATGGGGAGAGGCAGTGTG |  |  |
| [NM_003840.4](http://www.ncbi.nlm.nih.gov/entrez/viewer.fcgi?db=nucleotide&id=384871638) | TNFRSF10D (h) | Fw | CTGCGAGAACCTTTGCACG | 85 bp | 101.8% |
|  |  | Rv | GAGAAGGGAGGAGGGTGGAT |  |  |
| [NM_199168.3](http://www.ncbi.nlm.nih.gov/entrez/viewer.fcgi?db=nucleotide&id=291045298) | CXCL12 (h) | Fw | ATTCTCAACACTCCAAACTGTGC | 88 bp | 99.8% |
|  |  | Rv | ACTTTAGCTTCGGGTCAATGC |  |  |
