# Supplementary figures and images for: A role for human brain pericytes in neuroinflammation
Source: J Neuroinflammation. 2014 Jun 11;11:104. doi: 10.1186/1742-2094-11-104 (PMC4105169; doi:10.1186/1742-2094-11-104)

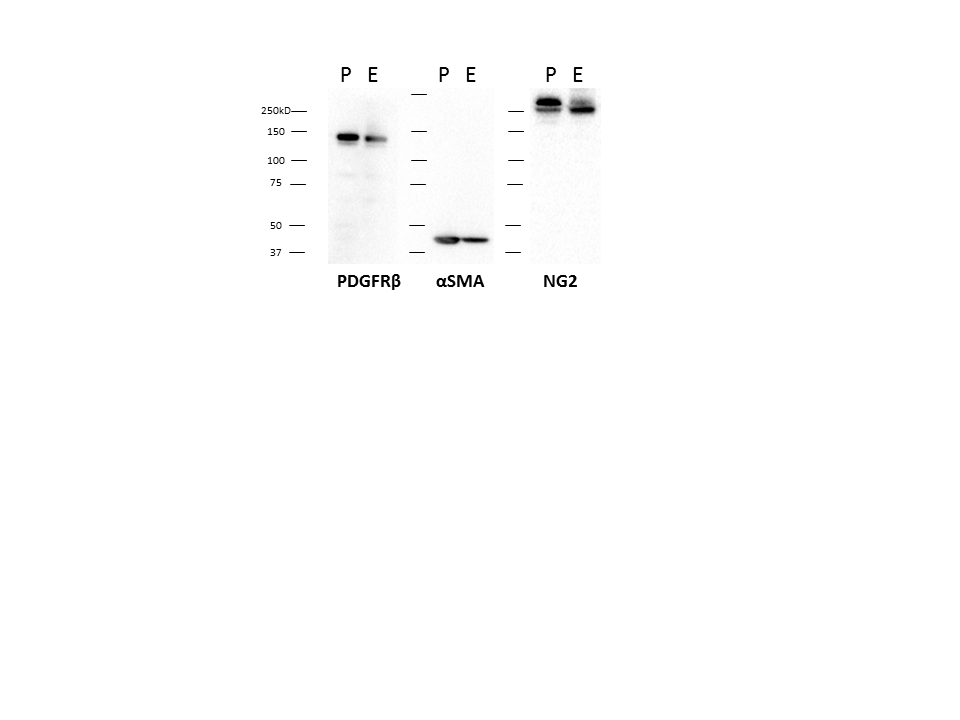

Supplement: Additional file 3: Figure S1 — Western blot analysis of pericyte culture extracts (P) and explant culture extracts (E) in untreated conditions confirming specificity of antibodies used for immunocytochemistry of αSMA, PDGFR-β and NG2. [file 1742-2094-11-104-S3.TIF]

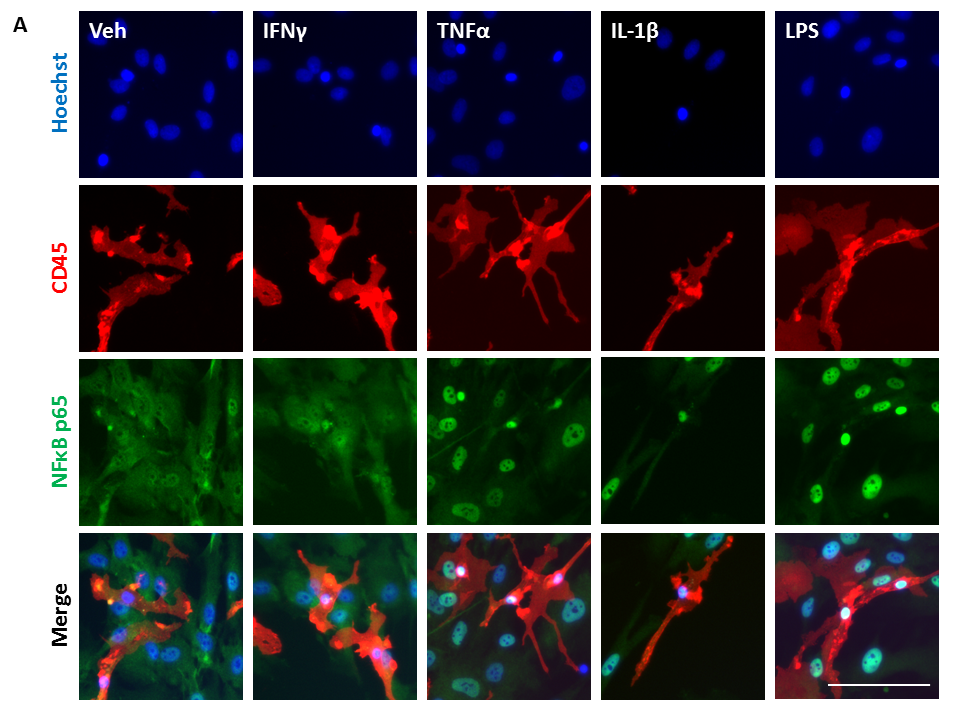

Supplement: Additional file 4: Figure S2 — NFκB p65 translocates to the nucleus after treatment with TNFα, IL-1β, and LPS in microglia from mixed glial cultures. Mixed glial cultures treated with vehicle (0.1% BSA in PBS), IFNγ (10 ng/ml), TNFα (50 ng/ml), IL-1β (10 ng/ml) or LPS for two hours then stained by immunocytochemistry for NFκB p65 (green), CD45 (red), and Hoechst (blue). Scale bar =100 μm. Images are representative of experiments done in triplicate, repeated in two separate cases. [file 1742-2094-11-104-S4.TIF]

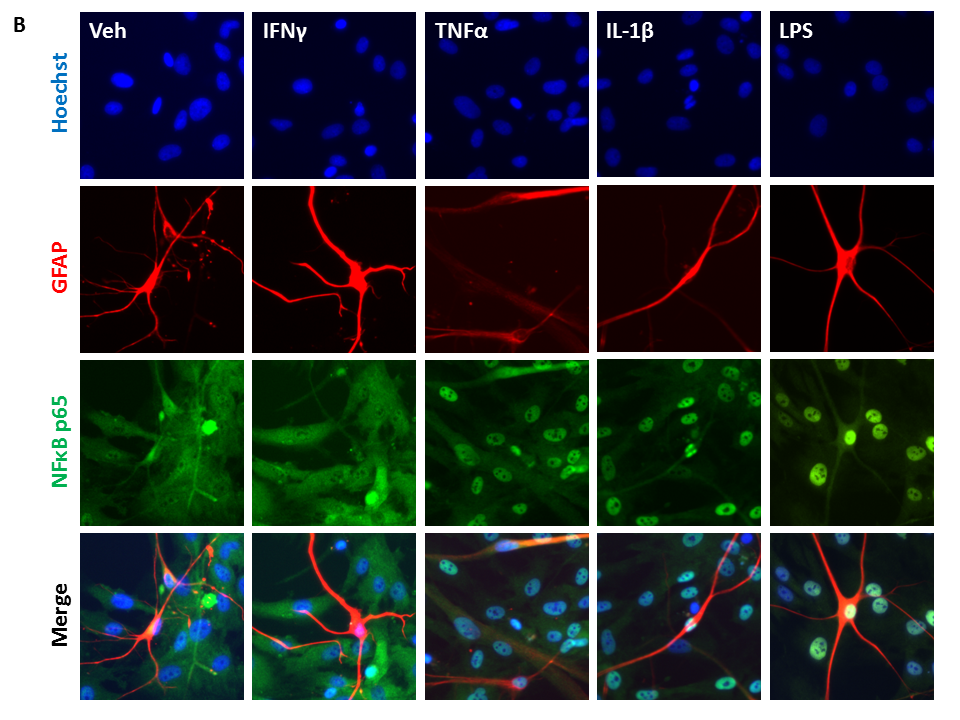

Supplement: Additional file 5: Figure S3 — NFκB p65 translocates to the nucleus after treatment with TNFα, IL-1β, and LPS in astrocytes from mixed glial cultures. Mixed glial cultures treated with vehicle (0.1% BSA in PBS), IFNγ (10 ng/ml), TNFα (50 ng/ml), IL-1β (10 ng/ml) or LPS for two hours then stained by immunocytochemistry for NFκB p65 (green), GFAP (red) and Hoechst (blue). Scale bar =100 μm. Images are representative of experiments done in triplicate, repeated in two separate cases. [file 1742-2094-11-104-S5.TIF]

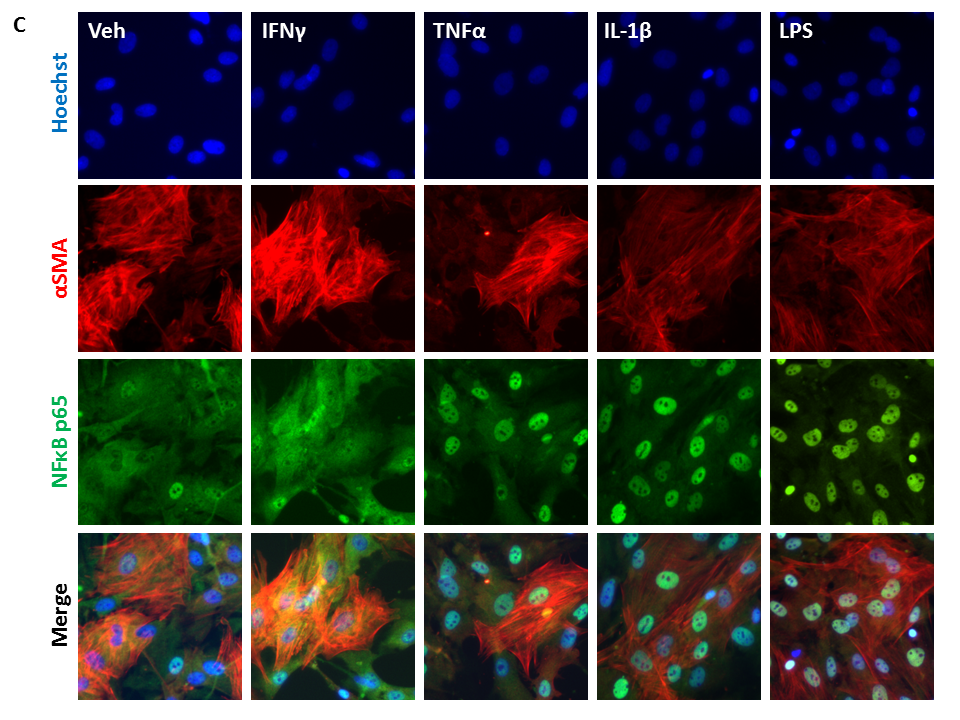

Supplement: Additional file 6: Figure S4 — NFκB p65 translocates to the nucleus after treatment with TNFα, IL-1β, and LPS in pericytes from mixed glial cultures. Mixed glial cultures treated with vehicle (0.1% BSA in PBS), IFNγ (10 ng/ml), TNFα (50 ng/ml), IL-1β (10 ng/ml) or LPS for two hours then stained by immunocytochemistry for NFκB p65 (green) and αSMA (red) and Hoechst (blue). Scale bar =100 μm. Images are representative of experiments done in triplicate, repeated in two separate cases. [file 1742-2094-11-104-S6.TIF]

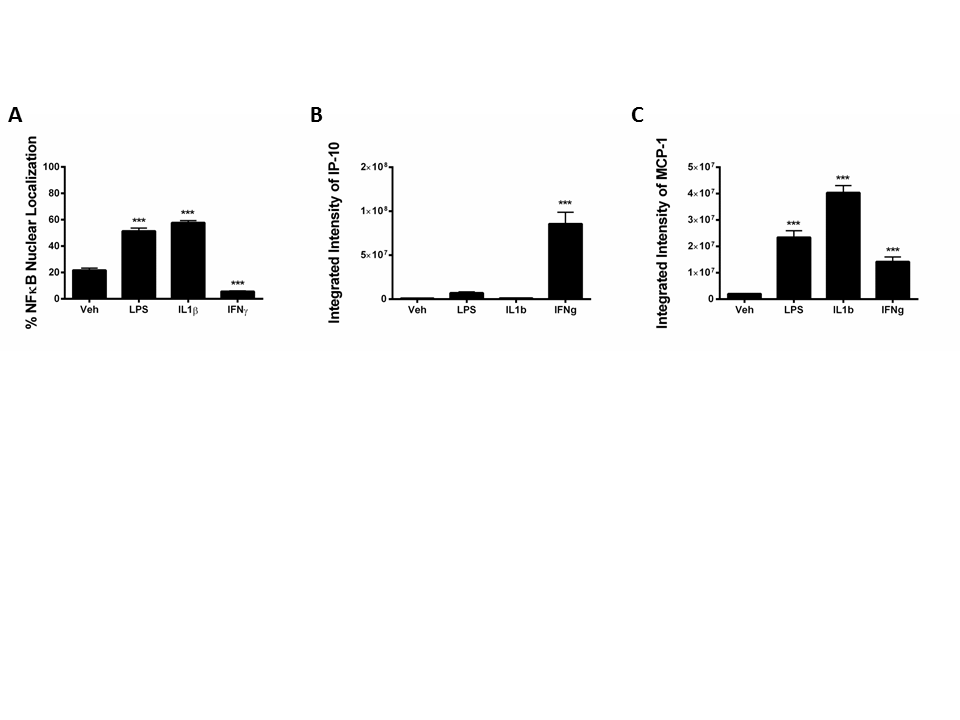

Supplement: Additional file 7: Figure S5 — Leptomeningeal explant cultures express IP-10 and MCP-1 and activate NFκB nuclear translocation in response to pro-inflammatory stimuli. A. Quantification of NFκB translocation in leptomeningeal explant cultures after two hours treatment with LPS (10 ng/ml), IL-1β (10 ng/ml), or IFNγ (10 ng/ml) labelled with NFκB p65. This was analyzed using Metamorph Image Analysis software (nuclear translocation assay). B. Quantification of IP-10 staining intensity in cells immunocytochemically positive for IP-10 expression after 24 hours treatment with LPS (10 ng/ml), IL-1β (10 ng/ml), or IFNγ (10 ng/ml). C. Quantification of MCP-1 staining intensity in cells immunocytochemically positive for MCP-1 expression after 24 hours treatment LPS (10 ng/ml), IL-1β (10 ng/ml), or IFNγ (10 ng/ml). Analysis is representative of experiments repeated in at least three cases. [file 1742-2094-11-104-S7.TIF]

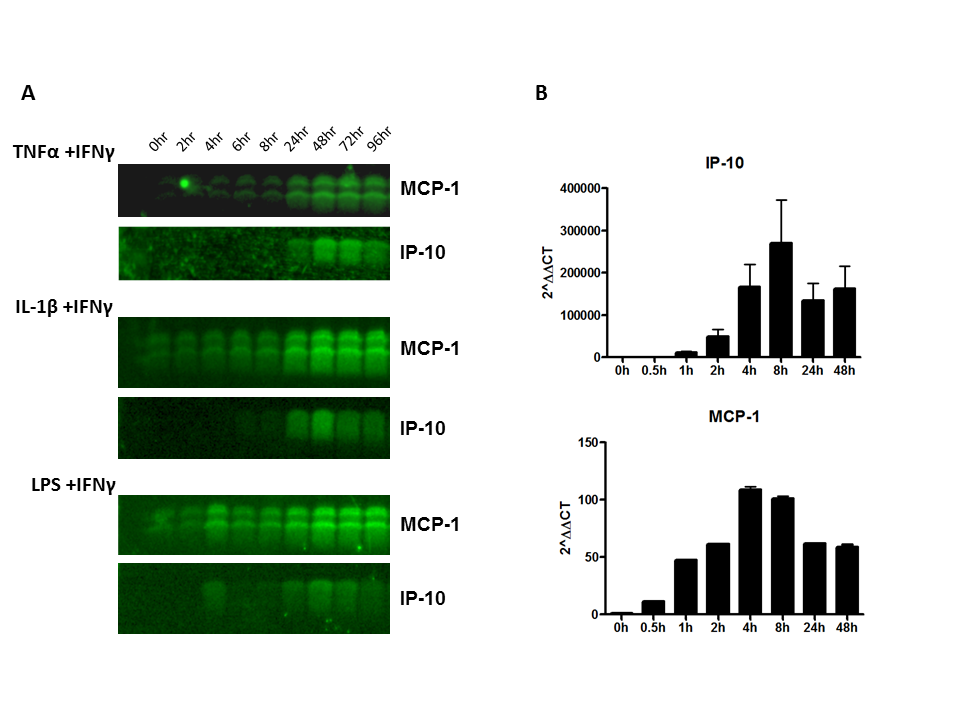

Supplement: Additional file 8: Figure S6 — IP-10 and MCP-1 mRNA expression and protein secretion increases in a time-dependent manner in human brain pericyte cells. A. Western blot analysis of IP-10 and MCP-1 secretion in conditioned media from late passage primary brain cells in response to combination treatment of TNFα (50 ng/ml), IL-1β (10 ng/ml) and LPS (10 ng/ml) with IFNγ (10 ng/ml). Western blot is representative of experiment repeated in two separate cases. B. qRT-PCR time-course analysis measuring IP-10 and MCP-1 gene expression in primary brain cells treated with IFNγ (10 ng/ml) and IL-1β (10 ng/ml). Data are representative of experiment repeated in two separate cases. [file 1742-2094-11-104-S8.TIF]

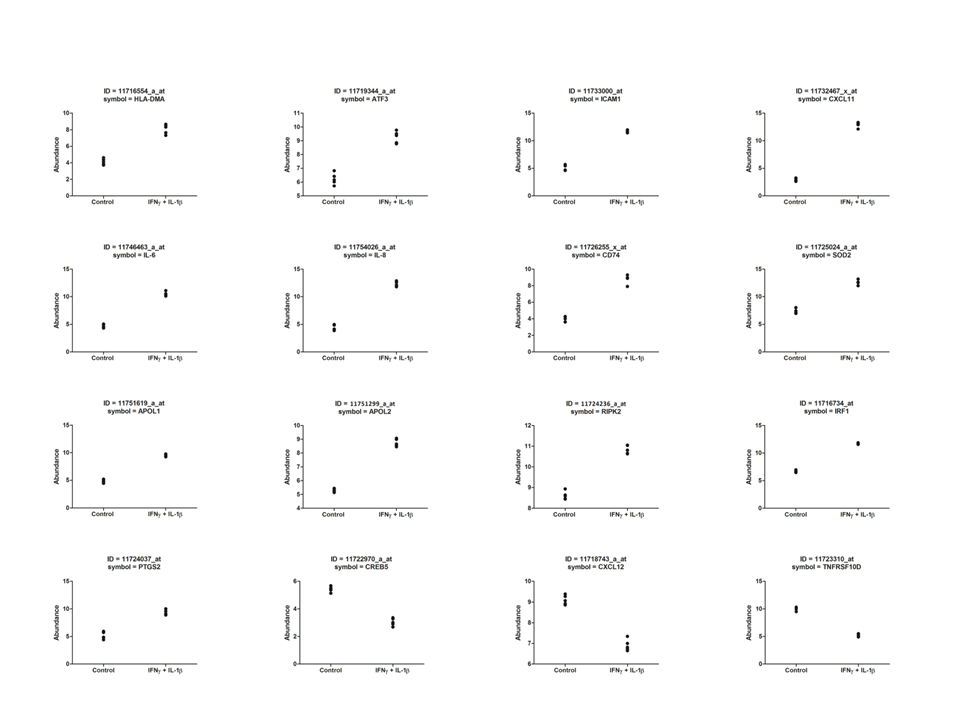

Supplement: Additional file 9: Figure S7 — Analysis of raw data from microarray experiment of genes chosen for validation by qRT-PCR reveals significant changes in gene expression. Each graph represents raw data from microarray experiments for the genes selected for confirmation by qRT-PCR. Each point is the expression of that gene by each patient sample in the designated condition (Vehicle (Veh) or IFNγ + IL-1β treated). [file 1742-2094-11-104-S9.TIF]

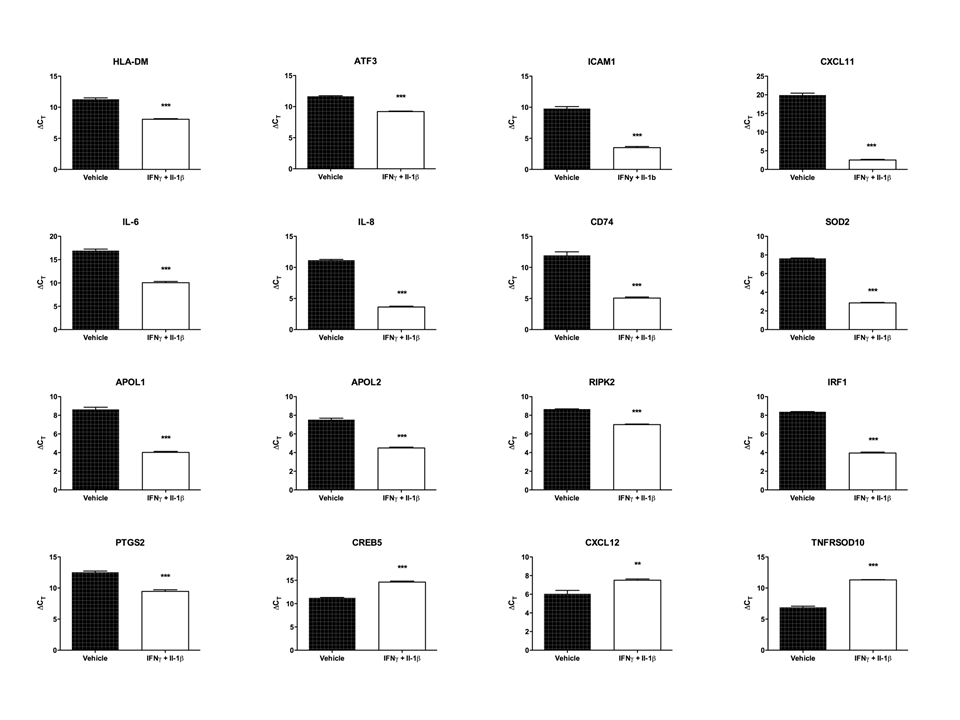

Supplement: Additional file 10: Figure S8 — Analysis of ΔCt values from qRT-PCR validation experiments reveals significant changes in gene expression across passages. Each graph represents mean ΔCt values for vehicle or IFNγ + IL-1β treated cultures. This experiment was repeated three times in one case over three successive passages. [file 1742-2094-11-104-S10.TIF]
